# Design and Optimization of an Anthropomorphic Robot Finger

**DOI:** 10.3390/biomimetics10030170

**Published:** 2025-03-11

**Authors:** Ming Cheng, Li Jiang, Ziqi Liu

**Affiliations:** The State Key Laboratory of Robotics and System, Harbin Institute of Technology, Harbin 150008, China; mingcheng@hit.edu.cn (M.C.); liuzqcn@hit.edu.cn (Z.L.)

**Keywords:** robotic finger, underactuation, coupled-adaptive mechanism, parametric optimization

## Abstract

The coupled-adaptive underactuated finger offers two motion modes: pre-grasping and self-adaptive grasping. It can execute anthropomorphic pre-grasp motions before the proximal phalanx contacts an object and transitions to adaptive enveloping once contact occurs. The key to designing a coupled-adaptive finger lies in its configuration and parameter, which are crucial for achieving a more human-like design for the prosthetic hand. Thus, this paper proposes a configuration topology and parameter optimization design method for a three-joint coupled-adaptive underactuated finger. The finger mechanism utilizes a combination of prismatic pairs and a compression spring to facilitate the transition between coupled motion and adaptive motion. This enables the underactuated finger to perform coupled movements in free space and adaptive grasping motions once it makes contact with an object. Furthermore, this paper introduces a finger linkage parameter optimization method that takes the joint motion angles and overall dimensions as constraints, aiming to linearize the joint coupling motion ratios as the primary optimization objective. The design method proposed in this paper not only presents a novel linkage mechanism but also outlines and compares its isomorphic types. Furthermore, the optimization results provide an accurate maximum motion value for the finger.

## 1. Introduction

The exceptional manipulation abilities of human hands are not only reflected in their dexterity, but also in their mechanical compliance [1]. The human hand elastic drive system, consisting of bones, joints, and ligaments, can not only generate the desired motion trajectory but also automatically adjust the applied force and stiffness in response to changes in the surrounding environment and the object being manipulated. This adaptability is the key factor enabling the human hand to perform dexterous, stable, and compliant operations [2]. As a functional rehabilitation aids for amputees, the prosthetic hands are designed to restore their grasping and manipulative abilities [3]. Compared to general-purpose robotic hands, prosthetic hands demand a higher level of anthropomorphic design in terms of size, weight, control complexity, and motion patterns. Designing a prosthetic hand mechanism to reproduce the operation function of a human hand, the commonly used design philosophies can be summarized as follows:

(1) Fewer Actuators. The complexity of an anthropomorphic prosthetic hand’s control system is directly proportional to the number of actuators it employs. A higher number of actuators implies more complex control inputs, whereas fewer actuators reduce the control difficulty for the mechanical system. To help individuals with disabilities make better use of the limited myoelectric signals from their residual limbs, it is crucial to use as few actuators as possible in a mechanical prosthetic hand. Reducing the number of actuators not only lowers control complexity but also decreases the weight and overall cost of the hand [4]. (2) High Integration. It typically adopts a highly integrated mechatronic design approach that combines mechanical and control systems, leading to a more compact internal layout, smaller overall size, and lighter weight [5]. Furthermore, the anthropomorphic appearance and dimensions align closely with ergonomic requirements, enhancing both comfort and usability while also supporting modular design for easier maintenance and upgrades [6,7,8,9,10].

(3) Modularization. Modular design effectively reduces the weight of the prosthetic hand structure, enhances the interchangeability of parts, reduces manufacturing costs, and simplifies assembly complexity [11,12,13].

(4) Mechanical compliance. A transmission system with mechanical adaptability can quickly adjust the mechanism configuration to adapt to the grasped object without the intervention of sensing and feedback control. Mechanical compliance can effectively increase the level of mechanical intelligence of the prosthetic hand and improve the passive response ability of the prosthetic hand to the manipulated object [14,15,16,17].

(5) Extensive range of motion. A sufficiently large motion range can effectively enhance the grasping capability of the prosthetic hand [18,19].

To date, the prosthetic hands have primarily employed underactuated mechanisms as the core component of their transmission systems to achieve the above features [20,21,22]. The underactuated mechanism leverages the energy conversion characteristics of energy storage elements, endowing prosthetic hands with an embedded mechanically intelligent structure. This enables the prosthetic hand to quickly respond to external environmental disturbances and achieve adaptive enveloping grasps of objects at the hardware level without the need for intervention from the control system. However, due to the high degree of freedom, compact structure, and complexity of the human hand, most mechanical hands cannot perfectly replicate its functions. To address these challenges, researchers have explored the following approaches in prosthetic hand system design:

(1) Independent design of the thumb and modular design of the other four fingers. The thumb is designed separately due to its more complex trapezium bone structure compared to the other four fingers. Whether grasping, lifting, pushing, pulling, or supporting objects, multiple degrees of freedom in the thumb are required for these actions. As a result, most anthropomorphic prosthetic hands feature a uniquely designed thumb, where the abduction–adduction and flexion–extension motions are independently driven by separate motors. The other four fingers, in contrast, are designed as modular units with identical structures and functions [23,24].

(2) Reduce the degrees of freedom of the metacarpophalangeal joint (MCP joint) of the fingers. The MCP joint of the human hand is a dual-degree-of-freedom system. In addition to the bending degree of freedom, it also has the lateral freedom degree, making the operating space of the human hand fingers a three-dimensional space. In order to simplify the structure of the prosthetic hand, enhance the reliability of the prosthetic hand, and reduce the cost of the prosthetic hand, the MCP joint of the prosthetic hand mainly retains the bending degree of freedom while omitting the lateral freedom degree, retaining the configuration of three knuckles and three joints [25].

(3) Coupling-adaptive design: Human fingers can quickly approach the grasped object through pre-grasping movements in free space and then adjust the muscles to complete the grasping operation of complex-shaped objects. The pre-grasping refers to the proportional coupling of the three joints in human or robotic fingers before it makes contact with an object. In the design of underactuated fingers, this pre-grasp mode [26] is also called cage-grasp [27] or coupling-adaptive grasp [28,29], which has three advantages: (a) it can simulate the pre-bending movement of human fingers; (b) it can approach the manipulated object more quickly and complete the bending movement; (c) it can enable the underactuated fingers to effectively constrain the grasped object within the operating space, making it impossible for them to escape the closed space created by the underactuated prosthetic hand, and then adjust the finger configuration and joint angle until a stable grasp of the manipulated object is completed.

Despite the fact that lots of efficient attempts and efforts have been made for the prostheses design, indeed, it is a challenge to effectively integrate coupling-adaptive mechanisms into prosthetic hands with an anthropomorphic size, ensuring both robust pre-grasp and self-adaptive functionalities while maintaining a compact and reliable structure. Therefore, this paper proposes a novel compact design method for a coupled-adaptive finger transmission chain. To enable the finger to simultaneously perform coupled motion and adaptive decoupling motion, the study focuses on three key elements: the position of the driving linkage, the position of the decoupling joint, and the placement of elastic components. The design of the spring is a crucial element in underactuated finger mechanisms [30]. In linkage-based underactuated fingers, the position of the driving link directly affects the driving force and the complexity of the integrated design. Additionally, the most directly influential joint affecting the underactuated motion pattern is the decoupling joint. Therefore, for the coupled-adaptive mechanism proposed in this paper, these three aspects have been chosen as the core design criteria. The traditional cross-coupled four-bar mechanism can execute humanoid coupled motion without adaptive capacities [31]. On this basis, a topological analysis of the traditional cross-coupled four-bar mechanism is conducted to design an underactuated finger mechanism capable of replicating the grasping functionality of a human hand. Furthermore, a statistical method is employed to optimize the mechanism’s dimensional parameters. Using the initial and final positions of the finger configuration as boundary conditions, the optimization aims to linearize the transmission relationship between joint angles. This ensures that the underactuated finger can reach its absolute final position while maintaining effective performance.

This research expands on our previous work [32,33]. But there exist some significant differences. The previous works primarily focused on introducing stability analysis methods for coupled-adaptive fingers and biomechatronic integration design approaches for prosthetic hands. The main contributions of this paper are as follows: (1) Proposing a topological design method for coupled-adaptive linkage mechanisms, accompanied by a comparative analysis of this class of isomorphic fingers mechanisms; (2) Developing a parameter optimization design method for this finger linkage mechanism, using initial and final positions as constraint conditions.

The rest of this article is organized as follows. First, Section 2 elaborates the mechanism design of the coupled-adaptive finger. Then, Section 3 introduces the parameter optimization design method for this finger linkage mechanism. Finally, Section 4 concludes this article.

## 2. Coupled-Adaptive Finger Mechanism Design

The transmission systems of underactuated fingers commonly use mechanisms such as cables [34], gears [35], and linkages [36]. Cable-driven underactuated systems typically incorporate pulleys paired with elastic components as decoupling mechanisms for adaptive motion. The reverse motion of the finger is achieved through the potential energy stored in the elastic elements. The main advantage of the cable mechanism lies in its flexibility, enabling more adaptable motion transmission within the finger. However, its drawbacks include the difficulty of securing the cable, as it tends to slip off the drive or guide pulleys. This often necessitates the use of complex limiters and pre-tensioning mechanisms [37,38], resulting in reduced reliability. Gear mechanisms in underactuated prosthetic hand transmission systems offer smooth motion transmission but require significant space, entail high manufacturing costs, and demand precise assembly and alignment. These factors limit their suitability for integrated designs in anthropomorphic prosthetic hands. In comparison, linkage mechanisms generally employ geometrically constrained lower-pair joints, which provide surface contact, lower contact pressure, higher load capacity, and ease of manufacturing. Furthermore, linkage mechanisms offer high stiffness, bidirectional driving, and ease of achieving predefined motion trajectories [39,40]. In summary, this paper adopts planar linkage mechanisms to design the mechanical transmission system for underactuated fingers.

**Figure 1:**
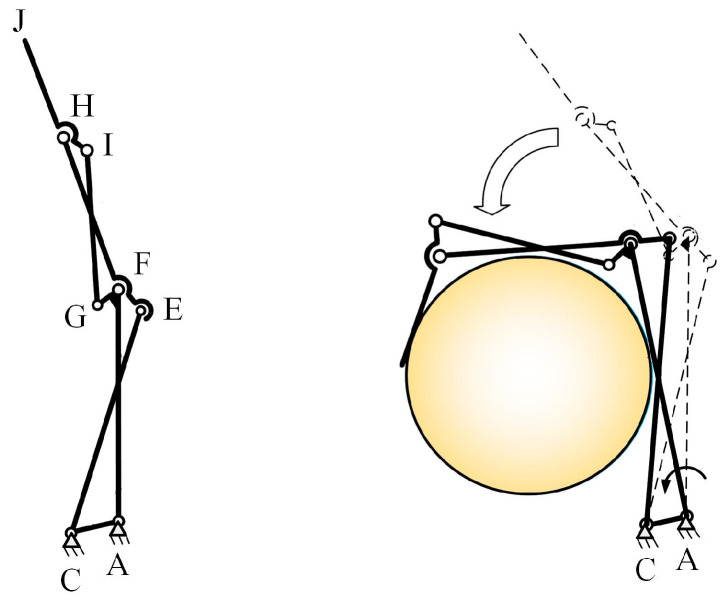
Structure of the coupled mechanism. The joints A, F, and H correspond to the MCP joint, PIP joint, and DIP joint, respectively. The dash line indicates the initial position.

The planar four-bar mechanism is often used to design adaptive anthropomorphic fingers [41]. The cross-coupled four-bar mechanism is commonly chosen for designing coupled fingers [42]. As shown in Figure 1, traditional coupled finger designs usually place the driving source at the metacarpophalangeal (MCP) joint. The actuators directly drive the rotation of the proximal phalanx (AF), while the coupled linkages enable the other phalanges to execute anthropomorphic pre-grasping motions. Therefore, this study uses the cross-coupled four-bar mechanism as the basic unit for type synthesis to design a novel underactuated finger structure, meeting the functional and adaptive requirements of underactuated prosthetic hands.

The type synthesis of mechanisms involves arranging and combining a specified number of components and kinematic pairs based on the required degrees of freedom. This process generates various possible mechanism types, from which the optimal topology is selected by analyzing and identifying the one that best meets the design objectives.

**Figure 2:**
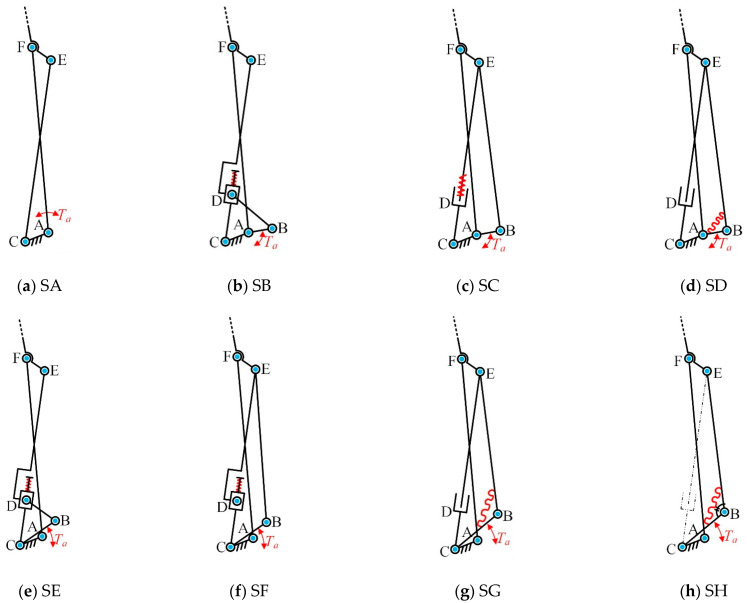
Type synthesis of linkage mechanism. *T*_a_ represents the driving position.

To enable the finger to achieve adaptive decoupling motion, this study focuses on three key elements: the position of the driving linkage, the location of the decoupling joint, and the placement of the elastic components. These three elements form the core of the underactuated coupled-adaptive finger mechanism. The traditional cross-coupled four-bar mechanism (Figure 2a) is deconstructed and reconfigured to create new topological structures. These structures enable underactuated finger transmission systems to perform both coupled and adaptive motion modes, as shown in Figure 2b–h. Among these, Figure 2b–g represent a seven-bar kinematic chain, while Figure 2h represents a five-bar kinematic chain. To clearly describe the different types of topological structures, the corresponding topologies shown in Figure 2 are labeled with the bold identifiers SA to SH throughout the text.

- The SA topological structure represents the traditional configuration of a cross-coupled four-bar mechanism.
- The SB topological structure is created by embedding a prismatic joint (D) into the coupling linkage (CE) of the SA topological structure. This modification allows the coupling four-bar mechanism to perform decoupled motions under specific conditions. Building on this, a closed kinematic chain (A-B-D-C-A) is added, forming a composite joint (D) that combines a prismatic joint and a revolute joint. An elastic component is installed at the composite joint (D), and the driving linkage is relocated from link AF to link AB. This creates a new seven-bar mechanism capable of performing coupled-adaptive motion.
- The SC topological structure is developed from the SB structure by merging joint D and joint E into a composite joint, resulting in a new seven-bar mechanism. Compared to the SB structure, the composite joint E in the SC structure combines two revolute joints and is simpler to implement structurally.
- The SD topological structure is an isomorphic variant of the SC structure. The key difference is the placement of the elastic component, which is located at joint B in the SD structure. Prismatic joints are generally suitable for linear elastic components, such as tension–compression elements, while revolute joints are better suited for torque-based elastic components. Therefore, different topological structures can be chosen based on specific design requirements.
- The SE topological structure is derived from the SB structure by relocating the driving position from joint A to joint C.
- The SF topological structure is based on the SE structure, with joint D and joint E merged into a composite joint.
- The SG topological structure is based on the SF structure, by relocating the elastic component from the prismatic joint D to joint B.
- The SH topological structure simplifies the SG structure by removing the adaptive linkage CE and adding a mechanical limiter at joint B to replace the coupling constraint function of linkage CE. This results in a new five-bar mechanism, achieving a more streamlined design.

Additionally, the elastic component in the SB~SH topological structures can be repositioned to joint F, forming new isomorphic variants labeled SBI~SHI. These variants are not illustrated in this paper for brevity.

As shown in Table 1, the SB~SH topological structures can be categorized into three groups for discussion:

(1) Position of the Driving Linkage

In the SB–SD topology, the driving link AB rotates around joint A, whereas in the SE–SH topology, the driving link CB rotates around joint C. The key difference lies in the behavior during the coupled motion phase, which occurs before the adaptive motion begins. In the SB–SD structures, the angular displacement of the revolute joint B starts to increase, while the translational offset of the prismatic joint D remains unchanged. In contrast, in the SE–SH structures, both the angular displacement of joint B and the translational offset of joint D remain constant.

As a result, the elastic component in the SE–SH structures, whether installed at joint B or D, maintains a constant spring force before adaptive motion begins. Additionally, having the driving linkage rotate around joint A in the SB–SD structures is advantageous for reducing the thickness of the finger, which facilitates the integrated design of the finger mechanism.

(2) Position of the Composite Joint

In the SC, SD, SF, SG, and SH topological structures, the force transmission point is located at the composite joint E, whereas in the SB and SE structures, the transmission point is at the composite joint D. Comparing these two categories, the pressure angle of the transmission linkage BE and the linkage CE in the SB and SE structures is larger, while the transmission angle is smaller. Conversely, the SC, SD, SF, SG, and SH structures feature a smaller pressure angle and a larger transmission angle between the transmission rod BD and the linkage CE. Therefore, the SB and SE topologies are more advantageous for efficient force transmission between the mechanism components.

(3) Spring Placement

In the SB, SC, SE, and SF topological structures, the elastic components are installed at the prismatic joint D. In contrast, the SD, SG, and SH structures have the elastic components installed at the revolute joint B. Elastic components placed at the prismatic joint D exhibit no compression variation during the coupled motion phase, resulting in better mechanical performance with zero energy loss. This configuration is also beneficial for the integrated design of the finger. For the SBI~SHI topological structures, the elastic components are relocated from joints B or D in the SB~SH structures to joint F, providing another variation for specific design requirements.

Based on the above analysis, this study utilizes the SB topological structure to design the transmission systems for the MCP and PIP joints of the anthropomorphic finger, enabling coupled-adaptive functionality. To simplify the finger’s design, the SA cross-coupled four-bar mechanism is applied to the PIP and DIP joints, achieving anthropomorphic coupled motion between them.

**Figure 3:**
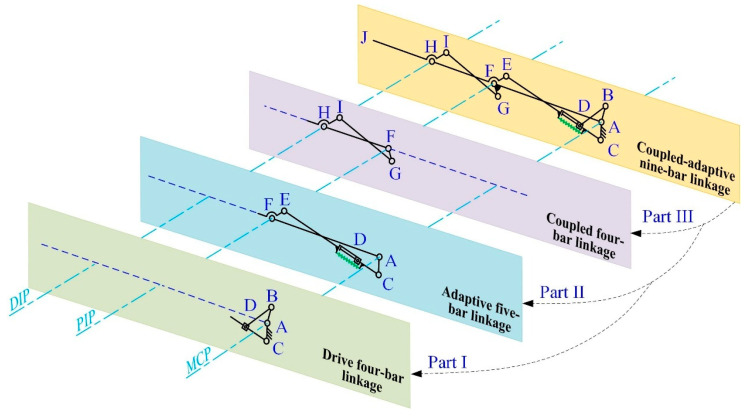
Composition of the nine-bar linkage mechanism. Part I is four-bar driving mechanism, Part II is five-bar adaptive mechanism, and Part III is four-bar coupling mechanism.

The coupled-adaptive nine-linkage mechanism, which integrates the SA and SB topological structures, is shown in Figure 3. Based on its linkage composition and mechanical motion transmission, the mechanism can be decomposed into three closed kinematic chains: the four-bar driving mechanism (A-B-D-C), the five-bar adaptive mechanism (A-C-D-E-F), and the four-bar coupling mechanism (F-G-I-H). First, the four-bar driving mechanism and the five-bar adaptive mechanism are connected in parallel, forming a coupled-adaptive seven-bar mechanism, where composite joint D integrates both prismatic and revolute motions. Then, the coupled-adaptive seven-bar mechanism is connected in series with the four-bar coupling mechanism, resulting in a coupled-adaptive nine-bar mechanism. This hybrid system combines serial and parallel linkages. Due to the presence of elastic joint D, the anthropomorphic finger based on this nine-linkage mechanism outputs two degrees of freedom only under specific external disturbances.

**Figure 4:**
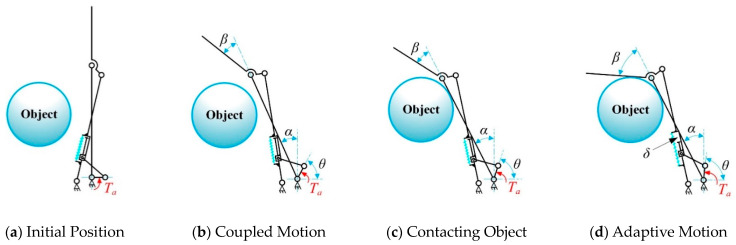
Sequence of the coupled-adaptive motion.

In Figure 3, hinge A, hinge F, and hinge H correspond to the MCP, PIP, and DIP joints of the anthropomorphic finger, respectively. Links AF, FH, and HJ represent the proximal, middle, and distal phalanges of the finger. Link AC is a fixed linkage attached to the base joint, AB is the driving linkage connected to the driving source, and BD is the follower link that transmits motion and force. The adaptive linkage group CE is composed of a crank CD and a sliding linkage DE, connected via the prismatic joint D. An elastic component is installed between CD and DE with a pre-compression force, maintaining the stability of the finger configuration in free space and preventing undesired motion before the proximal phalange (AF) contacts an object. In this state, the displacement of joint D remains zero (*δ* = 0), as shown in Figure 4a,b.

The compression spring also enables controlled decoupling motion during the adaptive phase. As illustrated in Figure 4c,d, once the proximal phalange (AF) contacts an object and stops moving, the PIP and DIP joints continue to move until the finger fully envelops the object. At this point, joint D generates a displacement offset *δ*, and the spring compression increases by *δ*. There is no adaptive behavior between the PIP and DIP joints; instead, their coupled motion is transmitted through the coupling rod GI. The joint angle transmission ratio between the DIP and PIP joints is designed as 2:3 [43], striking a balance between mimicking human grasping behavior and simplifying the complexity of the prosthetic hand.

## 3. Parameter Design of the Linkage Mechanisms

The previous section introduced the design of the coupled-adaptive nine-linkage mechanism with prismatic joints and elastic components, as shown in Figure 3. This mechanism is based on traditional coupling linkage structures and consists of three main parts: a four-bar driving mechanism, a five-bar adaptive mechanism, and a four-bar coupling mechanism. These parts serve three key functions: transmitting motion and force from the actuator to the finger phalanx, enabling coupled-adaptive motion of the MCP and PIP joints, and achieving coupled motion between the PIP and DIP joints.

Furthermore, this section employs the statistical mean square error method and uses the initial and final joint positions as design constraints to optimize the linkage parameters of the coupled-adaptive finger mechanism. The aim is to identify structural parameters for the transmission system that align with the requirements of integrated anthropomorphic prosthetic hand design.

**Figure 5:**
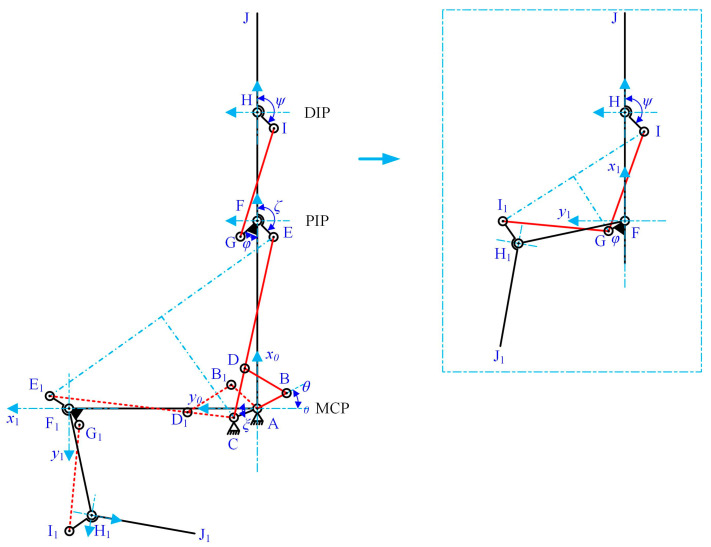
Geometric constraints in the optimal design of four-bar linkage. The maximum rotation angles of the MCP, PIP, and DIP joints are as follows: *α*_max_ = 90°, *β*_max_ = 102°, *γ*_max_ = 68°.

As shown in Figure 3, the coupled-adaptive nine-linkage mechanism can be divided into three kinematic chain subsystems: the coupled four-bar chain F-H-I-G-F, the adaptive five-bar chain A-F-E-D-C-A, and the driving four-bar chain A-B-D-C-A. As illustrated in Figure 5, in free space, the MCP and PIP joints, as well as the PIP and DIP joints, exhibit anthropomorphic coupled motion patterns. During this phase, the displacement *δ* of joint D remains zero. Thus, in free space, the kinematic chains A-F-E-D-C-A and F-H-I-G-F can be equivalently simplified into two isomorphic cross-coupled four-bar mechanisms A-F-E-C-A and F-H-I-G-F. Since motion transmission in non-parallelogram four-bar mechanisms is inherently nonlinear, the objective of subsequent parameter optimization is to achieve an approximately linear motion relationship between joint angles.

As shown in Figure 5, links AF, FH, and HJ correspond to the proximal, middle, and distal phalanges of the underactuated prosthetic finger, respectively. The angles are defined as follows: *θ* represents the driving angle of link AB; α represents the rotation angle of link AF relative to the base coordinate axis (MCP joint angular displacement); *β* represents the rotation angle of link FH relative to link AF (PIP joint angular displacement); and *γ* represents the rotation angle of link HJ relative to link FH (DIP joint angular displacement).

The coupled-adaptive linkage mechanism serves as the core component for transmitting motion and force in anthropomorphic fingers. Meanwhile it is one of the most complex structures in the mechanical replication of human fingers. Considering the requirements for anthropomorphic design and integrated functionality, the parameters of certain linkages in the coupled-adaptive mechanism can first be determined based on anthropomorphic design principles. Subsequently, the remaining linkage parameters can be calculated using geometric methods and the mean square error optimization approach, ensuring the mechanism’s kinematic relationships are accurately maintained.

### 3.1. Design of Finger Segment Lengths and Joint Angle Ranges

#### 3.1.1. Length of the Finger Segments

Underactuated prosthetic fingers must mimic the natural structure of human fingers in terms of appearance, dimensions, and range of motion. Based on our previous measurements and statistical analysis of human hand parameters [44], and considering practical design requirements such as reducing width and thickness to accommodate additional components like sensors and gloves, the dimensions of the three segments of the anthropomorphic finger are designed as follows: AF = 45 mm, FH = 26 mm, HJ = 25 mm.

#### 3.1.2. Width and Thickness of Finger Segments

The thickness and width of the finger segments are as follows: The proximal phalanx thickness *Tf*_pp_ ≤ 17 mm; The media phalanx thickness *Tf*_mp_ ≤ 15.5 mm; The distal phalanx thickness *Tf*_dp_ ≤ 15.5 mm; The finger joint width *Bf* ≤ 16 mm.

#### 3.1.3. Range of Motion of the Finger Joints

To ensure the underactuated finger achieves an anthropomorphic range of motion (ROM) and functionality, the motion ranges for the three joints are designed as follows: *α*_max_ = 90°, β_max_ = 102°, *γ*_max_ = 68°. Additionally, the transmission ratio between the DIP and PIP joints is set to 2:3, allowing the coupled motion relationship between the middle and distal phalanges of the human finger to be replicated [45].

### 3.2. Sequence of Parameter Optimization for the Linkage Mechanism

To minimize the number of design parameters requiring optimization, after determining basic parameters such as finger segment lengths and joint angle ranges, the following methods are applied: (a) In the A-F-E-C-A kinematic chain, determine the installation position of hinge E, specifically the length and angle *ζ* of link EF. (b) In the F-H-I-G-F kinematic chain, determine the installation position of hinge I, specifically the length and angle *ψ* of link HI. (c) Optimize the lengths of links AC, CE, FG, and GI to achieve a quasi-linear transfer ratio between the joint angles.

These steps ensure effective parameter determination while reducing the complexity of the optimization process. The solution sequence is as follows: First, solve the linkage parameters in the coupled four-bar kinematic chain F-H-I-G-F. Then, determine the parameters in the adaptive five-bar kinematic chain A-F-E-D-C-A. Finally, solve the linkage parameters in the driving four-bar kinematic chain A-B-D-C-A.

### 3.3. Optimization Design of Linkage Parameters Based on Geometric and Mean Square Error Methods

As shown in Figure 5, to achieve the anthropomorphic design of the underactuated finger, this study sets the boundary conditions such that the MCP, PIP, and DIP joints of the underactuated finger can reach their maximum rotational positions, *α*_max_, *β*_max_, and *γ*_max_, respectively, in a free space. Based on these conditions, a constraint relationship can be established between the linkages *GI* and *FG* as

(1) $$GI=F_{1}\left(FG,FH,HI,\psi \right),$$

A constraint relationship is established between the linkages *CE* and *AC* as

(2) $$CE=F_{2}\left(AC,AF,EF,\xi \right),$$

The boundary constraint condition expression (1) for *GI* and *FG* can be derived from Equations (3) and (4)

(3) $$GI=\sqrt{{\left(\frac{I_{y}-I_{1y}}{I_{1x}-I_{x}}G_{y}+\frac{I_{1x}^{2}+I_{1y}^{2}-I_{x}^{2}-I_{y}^{2}}{2I_{1x}-2I_{x}}-I_{x}\right)}^{2}+{\left(G_{y}-I_{y}\right)}^{2}},$$

(4) $$G_{x}=\frac{I_{y}-I_{1y}}{I_{1x}-I_{x}}G_{y}+\frac{I_{1x}^{2}+I_{1y}^{2}-I_{x}^{2}-I_{y}^{2}}{2I_{1x}-2I_{x}},$$

where *I_x_* and *I_y_* represent the horizontal and vertical coordinates of point I in the coordinate system {*x*_1_F*y*_1_}; *I*_1*x*_ and *I*_1*y*_ represent the horizontal and vertical coordinates of point I1 in the coordinate system {*x*_1_F*y*_1_}; and *G*_x_ and *G*_y_ represent the horizontal and vertical coordinates of point G in the coordinate system {*x*_1_F*y*_1_}.

Based on the geometric constraints shown in Figure 5, the relationships between *I_x_*, *I_y_*, *I*_1*x*_, *I*_1*y*_, and the joint angle ranges *α*_max_, *β*_max_, and *γ*_max_ can be derived as

(5) $$I_{x}=FH+HI\cos\psi ,$$

(6) $$I_{y}=-HI\sin\psi ,$$

(7) $$I_{1x}=FH\cdot \cos\beta_{\max}+HI\cdot \cos\left(\beta_{\max}+\gamma_{\max}-\psi \right),$$

(8) $$I_{1y}=FH\cdot \sin\beta_{\max}+HI\cdot \sin\left(\beta_{\max}+\gamma_{\max}-\psi \right),$$

Based on the finger thickness constraint, the constraint range for *G*(*G_x_*, *G_y_*) is set as *G_y_* ≤ *Tf*_mp_ ⁄2. Additionally, since F-H-I-G is a cross-coupled four-bar mechanism, *G_y_* ≥ 0. Combining these conditions, the constraint range for *G*(*G_x_*, *G_y_*) is

(9) $$0\leq G_{y}\leq \frac{Tf_{\mathrm{mp}}}{2},$$

The boundary constraint condition expression (2) for *CE* and *AC* can be derived from Equations (10) and (11) as

(10) $$CE=\sqrt{{\left(\frac{E_{y}-E_{1y}}{E_{1x}-E_{x}}C_{y}+\frac{E_{1x}^{2}+E_{1y}^{2}-E_{x}^{2}-E_{y}^{2}}{2E_{1x}-2E_{x}}-E_{x}\right)}^{2}+{\left(C_{y}-E_{y}\right)}^{2}},$$

(11) $$C_{x}=\frac{E_{y}-E_{1y}}{E_{1x}-E_{x}}C_{y}+\frac{E_{1x}^{2}+E_{1y}^{2}-E_{x}^{2}-E_{y}^{2}}{2E_{1x}-2E_{x}},$$

where *E_x_* and *E_y_* represent the horizontal and vertical coordinates of point *E* in the coordinate system {*x*_0_A*y*_0_}; *E*_1*x*_ and *E*_1*y*_ represent the horizontal and vertical coordinates of point E1 in the coordinate system {*x*_0_A*y*_0_}; *C_x_* and *C_y_* represent the horizontal and vertical coordinates of point C in the coordinate system {*x*_0_A*y*_0_}.

Based on the geometric constraints shown in Figure 5, the relationships between *E_x_*, *E_y_*, *E*_1*x*_, *E*_1*y*_, and the joint angle ranges *α*_max_, *β*_max_, and *γ*_max_ can be derived as

(12) $$E_{x}=AF+EF\cdot \cos\zeta ,$$

(13) $$E_{y}=-EF\cdot \sin\zeta ,$$

(14) $$E_{1x}=AF\cdot \cos\alpha_{\max}+EF\cdot \cos\left(\alpha_{\max}+\beta_{\max}-\zeta \right),$$

(15) $$E_{1y}=AF\cdot \sin\alpha_{\max}+EF\cdot \sin\left(\alpha_{\max}+\beta_{\max}-\zeta \right),$$

Based on the finger thickness constraint, the constraint range for *C*(*C_x_*, *C_y_*) is set as *C_y_* ≤ *Tf*_pp_ ⁄2. Additionally, since A-F-E-C is a cross-coupled four-bar mechanism, C*_y_* ≥ 0. Combining these conditions, the constraint range for *C*(*C_x_*, *C_y_*) is

(16) $$0\leq C_{y}\leq \frac{Tf_{\mathrm{pp}}}{2},$$

The advantage of constraining the relationships between *CE* and *AC*, as well as between *GI* and *FG*, using the maximum joint angle positions is that it ensures zero error between the maximum joint angle positions and the anthropomorphic target values. Additionally, it reduces the number of parameters that need to be optimized. Through the above constraints, the remaining parameters to be determined in the transmission linkage mechanism are simplified to the design positions *G_y_* for *G*(*G_x_*,*G_y_*) and *C_y_* for *C*(*C_x_*,*C_y_*).

Unlike the design requirement for maximum joint angle positions, where joint angle vectors are fixed, the design goal for the driving angle is to achieve linearized transmission as much as possible while meeting the adaptive size constraints. To further reduce the number of parameters requiring optimization, the initial installation position of the prismatic joint D (the value of CDi) in the linkage C-D-E is first determined in the A-B-D-C-A kinematic chain. Additionally, the lengths of the driving linkages AB and BD are specified. Consequently, the parameters to be optimized in the A-B-D-C-A kinematic chain are the values of *θ*_0_ and *θ*_max_. Based on the geometric relationships, the constraint conditions can then be designed as

(17) $$\left\{ \begin{array}{c} f_{\mathrm{ad}}\left(\alpha ,\beta_{\max}\right)<AB+BD\\ \;-22.5{}^{\circ}<\theta_{0}<22.5{}^{\circ}\\ \;90{}^{\circ}<\theta_{\max}<180{}^{\circ} \end{array},\right.$$

where *f*_ad_(*α*, *β*) is the Distance from hinge A to hinge D. In the process of calculating the connecting rod parameters, the relationship between the joint angles *δ*_1_, *δ*_2_, and *δ*_3_ is used as the main reference index

(18) $$\left[\begin{array}{c} \delta_{1}\\ \delta_{2}\\ \delta_{3} \end{array}\right]=\left[\begin{array}{c} \beta -{ƛ}_{1}\gamma \\ \alpha -{ƛ}_{2}\beta \\ \theta -{ƛ}_{3}\alpha \end{array}\right],$$

Based on the geometric relationships between the linkages illustrated in Figure 5, the precise analytical expression for the coupling relationship among the joint angles *α*, *β*, and *γ* can be derived as

(19) $$\begin{array}{ll} \beta & =\arccos\left(\frac{FH+HI\cos\left(\gamma -\psi \right)}{\sqrt{FH^{2}+HI^{2}+2FH\cdot HI\cos\left(\gamma -\psi \right)}}\right)+\pi -\varphi \\ & -\arccos\left(\frac{FG^{2}+FH^{2}+HI^{2}-GI^{2}+2FH\cdot HI\cos\left(\gamma -\psi \right)}{2FG\sqrt{FH^{2}+HI^{2}+2FH\cdot HI\cos\left(\gamma -\psi \right)}}\right) \end{array},$$

(20) $$\begin{array}{ll} \alpha & =\arccos\left(\frac{AF+EF\cos\left(\beta -\zeta \right)}{\sqrt{AF^{2}+EF^{2}+2AF\cdot EF\cos\left(\beta -\zeta \right)}}\right)+\xi +0.5\pi \\ & -\arccos\left(\frac{AC^{2}+AF^{2}+EF^{2}-{\left(CD_{i}+DE\right)}^{2}+2AF\cdot EF\cos\left(\beta -\zeta \right)}{2AC\sqrt{AF^{2}+EF^{2}+2AF\cdot EF\cos\left(\beta -\zeta \right)}}\right) \end{array},$$

(21) $$\begin{array}{ll} \theta & =-\arccos\left(\frac{AB^{2}+AC^{2}+CD_{i}^{2}-BD^{2}-2AC\cdot CD_{i}\cos X_{1}}{2AB\sqrt{AC^{2}+CD_{i}^{2}-2AC\cdot CD_{i}\cos X_{1}}}\right)\\ & -\arccos\left(\frac{AC-CD_{i}\cos X_{1}}{\sqrt{AC^{2}+CD_{i}^{2}-2AC\cdot CD_{i}\cos X_{1}}}\right)+\xi +\pi -\theta_{0} \end{array},$$

where

(22) $$\begin{array}{ll} X_{1} & =\arccos\left(\frac{AC+AF\sin\left(\xi -\alpha \right)}{\sqrt{AC^{2}+AF^{2}+2AC\cdot AF\sin\left(\xi -\alpha \right)}}\right)\\ & -\arccos\left(\frac{{\left(CD_{i}+DE\right)}^{2}+AC^{2}+AF^{2}-EF^{2}+2AC\cdot AF\sin\left(\xi -\alpha \right)}{2\left(CD_{i}+DE\right)\sqrt{AC^{2}+AF^{2}+2AC\cdot AF\sin\left(\xi -\alpha \right)}}\right) \end{array},$$

The parameters ${ƛ}_{1}$, ${ƛ}_{2}$, and ${ƛ}_{3}$ are the proportional coefficients for the designed maximum joint angles of the coupled finger, which can be expressed as:

(23) $$\left[\begin{array}{c} {ƛ}_{1}\\ {ƛ}_{2}\\ {ƛ}_{3} \end{array}\right]=\left[\begin{array}{c} \beta_{\max}/\gamma_{\max}\\ \alpha_{\max}/\beta_{\max}\\ \theta_{\max}/\alpha_{\max} \end{array}\right],$$

where *α*_max_ = 90° is the maximum motion angle of the MCP joint, *β*_max_ = 102° is the maximum motion angle of the PIP joint, and *γ*_max_ = 68° is the maximum motion angle of the DIP joint.

The optimization objectives for the linkage parameters are as follows: when the MCP joint angle *α* varies within the range of 0 to α_max_, the PIP joint angle *β* varies within the range of 0 to *β*_max_, and the DIP joint angle *γ* varies within the range of 0 to *γ*_max_, the statistical values *δ*_1_, *δ*_2_, and *δ*_3_ (including their means *E*(*δ*_1_), *E*(*δ*_2_), and *E*(*δ*_3_), and their variances *D*(*δ*_1_), *D*(*δ*_2_), and *D*(*δ*_3_)) should be minimized, as defined by Equations (24) and (25)

(24) $$E\left(\delta_{m}\right)=\sum_{k=1}^{n}\left|\frac{\delta_{\mathrm{mk}}}{n}\right|,\left(m\in \left\{1,2,3\right\}\right),$$

(25) $$D\left(\delta_{m}\right)=\sum_{k=1}^{n}\frac{{\left[\delta_{\mathrm{mk}}-E\left(\delta_{m}\right)\right]}^{2}}{n},\left(m\in \left\{1,2,3\right\}\right),$$

where *n* represents the number of sampling points for the joint angles within their respective motion ranges.

Based on the precision requirements in mechanical processing, the search accuracy for the installation positions of hinge C is set to 0.001 within the constraint range, while the search accuracy for the initial driving angle *θ*_0_ is set to 0.001° within its constraint range. Through iterative calculations, the computed values for the design parameters of the transmission linkage can be determined as

(26) $$\left\{ \begin{array}{c} E\left(\delta_{1}\right)=0.5471{}^{\circ}\\ D\left(\delta_{1}\right)=0.0013{}^{\circ}\\ \;G_{x}=-2.678\\ \;G_{y}=+2.766 \end{array},\right.$$

and

(27) $$\left\{ \begin{array}{c} E\left(\delta_{2}\right)=0.3378{}^{\circ}\\ D\left(\delta_{2}\right)=0.0007{}^{\circ}\\ \;C_{x}=-1.775\\ \;C_{y}=+5.886 \end{array},\right.$$

The computed values for the design parameters of the driving linkage are

(28) $$\left\{ \begin{array}{c} E\left(\delta_{3}\right)=0.2189{}^{\circ}\\ D\left(\delta_{3}\right)=0.0003{}^{\circ}\\ \;\theta_{0}=15.345{}^{\circ}\\ \;\theta_{\max}=148.00{}^{\circ} \end{array},\right.$$

**Figure 6:**
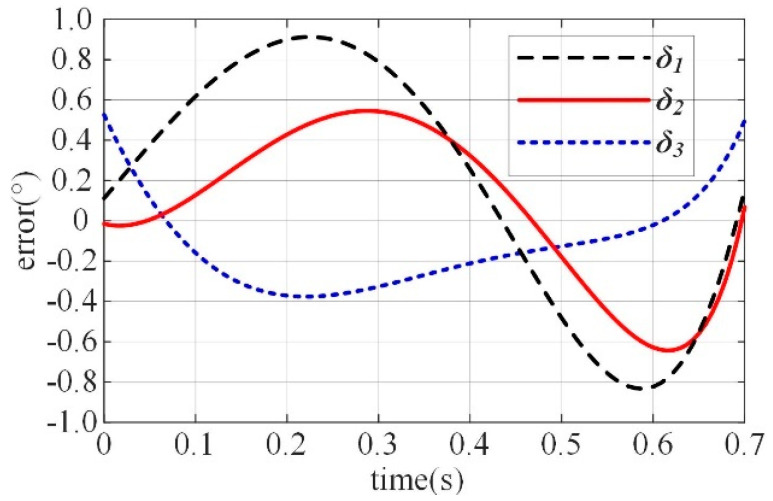
Linear errors of the joint angles.

The joint angle error *δ* curves are shown in Figure 6. The horizontal axis represents time, corresponding to the process of the finger moving from its initial position to the maximum designed position, while the vertical axis represents the linear error *δ* of the joint angles. From the figure, it can be observed that the maximum linear errors resulting from the design parameters are ${\left|\delta_{1}\right|}_{max}=0.923{}^{\circ},{\left|\delta_{1}\right|}_{max}=0.635{}^{\circ}$, and ${\left|\delta_{1}\right|}_{max}=0.531{}^{\circ}$. These results indicate that the design parameters meet the required precision criteria.

**Figure 7:**
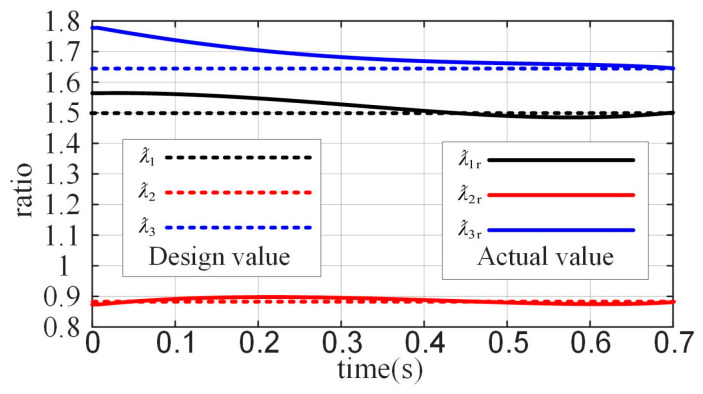
Joint angles coupling coefficient.

The coupling ratio coefficients ${ƛ}_{1}$, ${ƛ}_{2}$, and ${ƛ}_{3}$ for the joint angles are shown in Figure 7. The horizontal axis represents time, indicating the process of the finger moving from its initial position to the maximum designed position, while the vertical axis represents the values of the coupling ratio coefficients ${ƛ}_{1}$, ${ƛ}_{2}$, and ${ƛ}_{3}$. In the figure, the dashed lines represent the target coupling ratio coefficients ${ƛ}_{1}$, ${ƛ}_{2}$, and ${ƛ}_{3}$ at the maximum joint position, which correspond to the optimization target values. The solid lines represent the real-time coupling ratio coefficients ${ƛ}_{1r}$, ${ƛ}_{2r}$ and ${ƛ}_{3r}$. By combining these results with other predefined and derived parameter values, the design parameters for the coupled-adaptive linkage mechanism are summarized in Table 2 and Table 3. All length units are in millimeters.

## 4. Conclusions

The finger is the most fundamental execution unit for achieving dexterous and compliant manipulation tasks in prosthetic hands. This study focuses on a single modular underactuated finger mechanism and analyzes and compares homologous types of coupled-adaptive underactuated finger mechanisms, using a traditional cross-coupled lead screw mechanism as the topological analysis unit. Furthermore, the paper summarizes the characteristics of this class of underactuated fingers and proposes a design method for a coupled-adaptive nine-linkage mechanism suitable for humanoid fingers. The proposed three-joint finger mechanism incorporates multiple sets of linkage structures and utilizes linear motion pairs and compression springs to transition between coupled motion and adaptive motion. This design enables the underactuated finger to execute anthropomorphic coupled movements in free space and adaptive grasping motions when interacting with objects.

Moreover, by using the joint motion angles and dimensions of humanoid fingers as constraints and aiming to linearize the joint coupling motion ratios, a statistical approach was adopted to design the linkage parameters of the coupled-adaptive mechanism. The resulting optimized design achieved a maximum linear error of less than 1° for all three joint angles. This research provides a design basis for the integrated design of prosthetic hands. The design method proposed in this paper not only presents a novel linkage mechanism but also outlines and compares its isomorphic types. Furthermore, the optimization results provide an accurate maximum motion value for the finger. Future work will further investigate the integrated development and grasp planning of a five-fingered hand with coupled-adaptive fingers.

## Figures and Tables

**Table 1:** Comparison of different topological structures.

Topological Structure	Mechanism Type	Driving Joint Position	Compound Joint Position	Spring Installation Position
SB	Seven-bar mechanism	Joint A	Joint D: Prismatic + Revolute	Prismatic Joint D
SC	Seven-bar mechanism	Joint A	Joint E: Revolute + Revolute	Prismatic Joint D
SD	Seven-bar mechanism	Joint A	Joint E: Revolute + Revolute	Revolute Joint B
SE	Seven-bar mechanism	Joint C	Joint D: Prismatic + Revolute	Prismatic Joint D
SF	Seven-bar mechanism	Joint C	Joint E: Revolute + Revolute	Prismatic Joint D
SG	Seven-bar mechanism	Joint C	Joint E: Revolute + Revolute	Revolute Joint B
SH	Five-bar mechanism	Joint C	Joint E: Revolute	Revolute Joint B
SBI	Seven-bar mechanism	Joint A	Joint D: Prismatic + Revolute	Revolute Joint F
SCI	Seven-bar mechanism	Joint A	Joint E: Revolute + Revolute	Revolute Joint F
SDI	Seven-bar mechanism	Joint A	Joint E: Revolute + Revolute	Revolute Joint F
SEI	Seven-bar mechanism	Joint C	Joint D: Prismatic + Revolute	Revolute Joint F
SFI	Seven-bar mechanism	Joint C	Joint E: Revolute + Revolute	Revolute Joint F
SGI	Seven-bar mechanism	Joint C	Joint E: Revolute + Revolute	Revolute Joint F
SHI	Five-bar mechanism	Joint C	Joint E: Revolute	Revolute Joint F
SB	Seven-bar mechanism	Joint A	Joint D: Prismatic + Revolute	Prismatic Joint D

**Table 2:** Parameters of the links.

*Link*	*AF*	*FH*	*HJ*	*AB*	*AC*	*BD*	*CDi*	*DE*	*EF*	*FG*	*GI*	*HI*
***Length (mm)***	45	26	25	5.8	6.15	12	11.5	32.49	5.5	4.85	24.25	5.5

**Table 3:** Parameters of the positions.

*Position*	*ξ*	*φ*	*ζ*	*ψ*	*θ*_0_	*θ*max
***Angle***	0.093π	0.255π	0.75π	0.86π	0.085π	0.822π

## Data Availability

There are no data to be shared.
